# Supplementary material for: A Humanized Yeast Phenomic Model of Deoxycytidine Kinase to Predict Genetic Buffering of Nucleoside Analog Cytotoxicity
Source: Genes (Basel). 2019 Sep 30;10(10):770. doi: 10.3390/genes10100770 (PMC6826991; doi:10.3390/genes10100770)
Supplement: Supplementary file 1 [file genes-10-00770-s001.zip › Additional_Files/Additional_File7_GOTermHeatmaps.zip!/Additional_File7_GOTermHeatmaps/nucleotidyltransferase_activity.pdf]

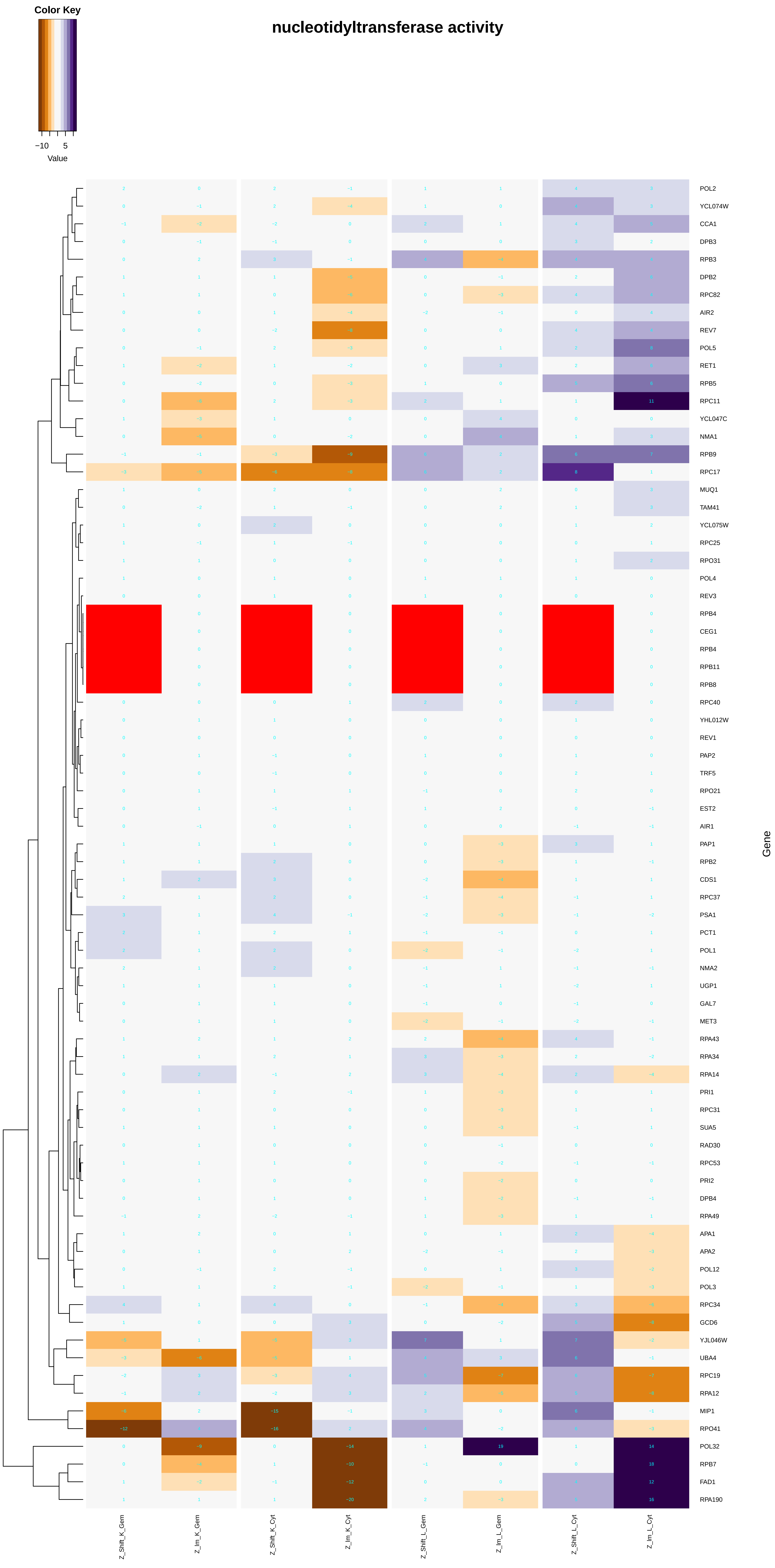
nucleotidyltransferase activity
Color Key
−10
5
Value
POL2
YCL074W
CCA1
DPB3
RPB3
DPB2
RPC82
AIR2
REV7
POL5
RET1
RPB5
RPC11
YCL047C
NMA1
RPB9
RPC17
MUQ1
TAM41
YCL075W
RPC25
RPO31
POL4
REV3
RPB4
CEG1
RPB4
RPB11
RPB8
RPC40
YHL012W
REV1
PAP2
TRF5
RPO21
EST2
AIR1
PAP1
RPB2
CDS1
RPC37
PSA1
PCT1
POL1
NMA2
UGP1
GAL7
MET3
RPA43
RPA34
RPA14
PRI1
RPC31
SUA5
RAD30
RPC53
PRI2
DPB4
RPA49
APA1
APA2
POL12
POL3
RPC34
GCD6
YJL046W
UBA4
RPC19
RPA12
MIP1
RPO41
POL32
RPB7
FAD1
RPA190
Gene
Z_Shift_K_Gem
Z_lm_K_Gem
Z_Shift_K_Cyt
Z_lm_K_Cyt
Z_Shift_L_Gem
Z_lm_L_Gem
Z_Shift_L_Cyt
Z_lm_L_Cyt

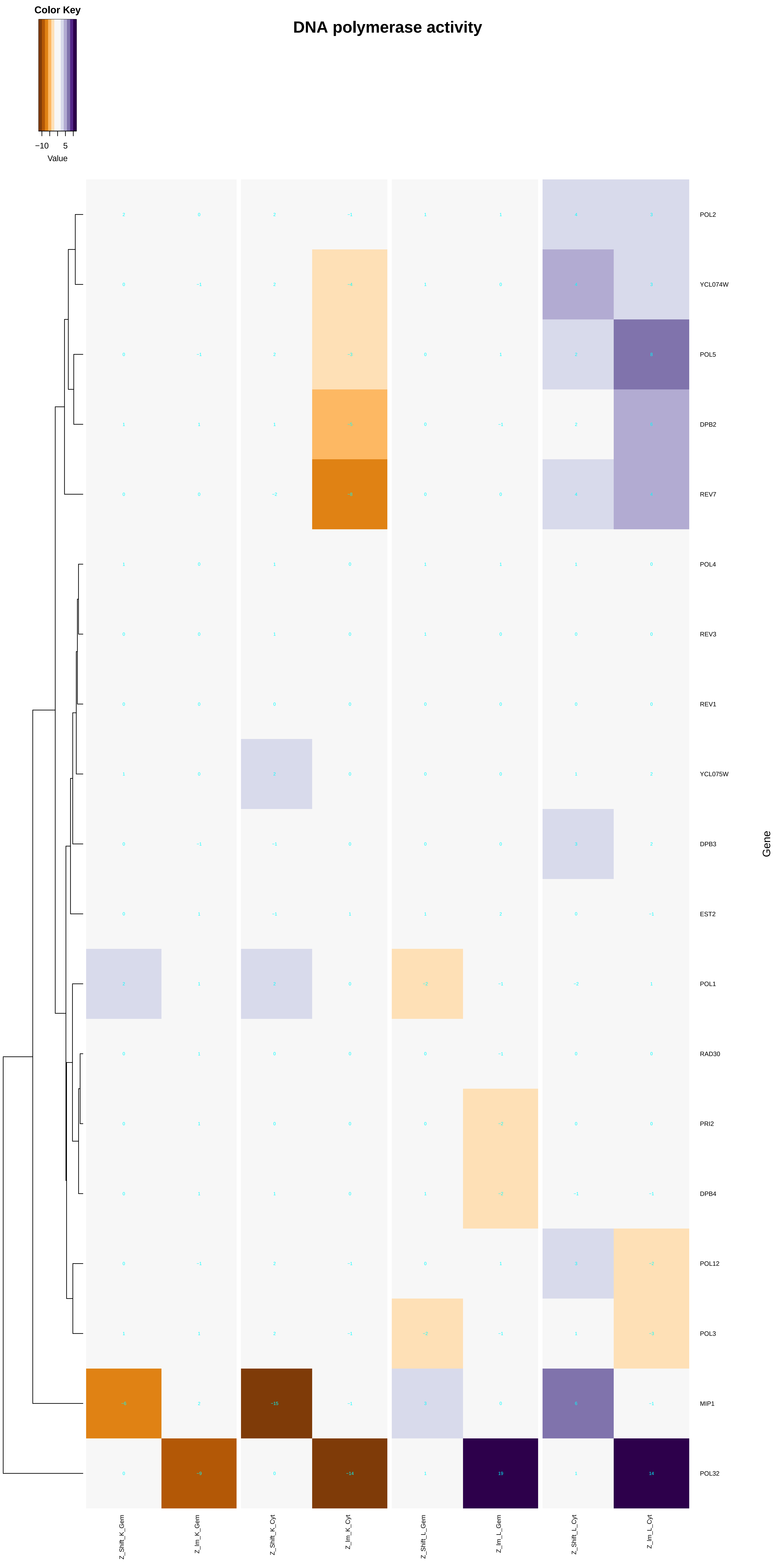
DNA polymerase activity
Color Key
−10
5
Value
POL2
YCL074W
POL5
DPB2
REV7
POL4
REV3
REV1
YCL075W
DPB3
EST2
POL1
RAD30
PRI2
DPB4
POL12
POL3
MIP1
POL32
Gene
Z_Shift_K_Gem
Z_Im_K_Gem
Z_Shift_K_Cyt
Z_Im_K_Cyt
Z_Shift_L_Gem
Z_Im_L_Gem
Z_Shift_L_Cyt
Z_Im_L_Cyt

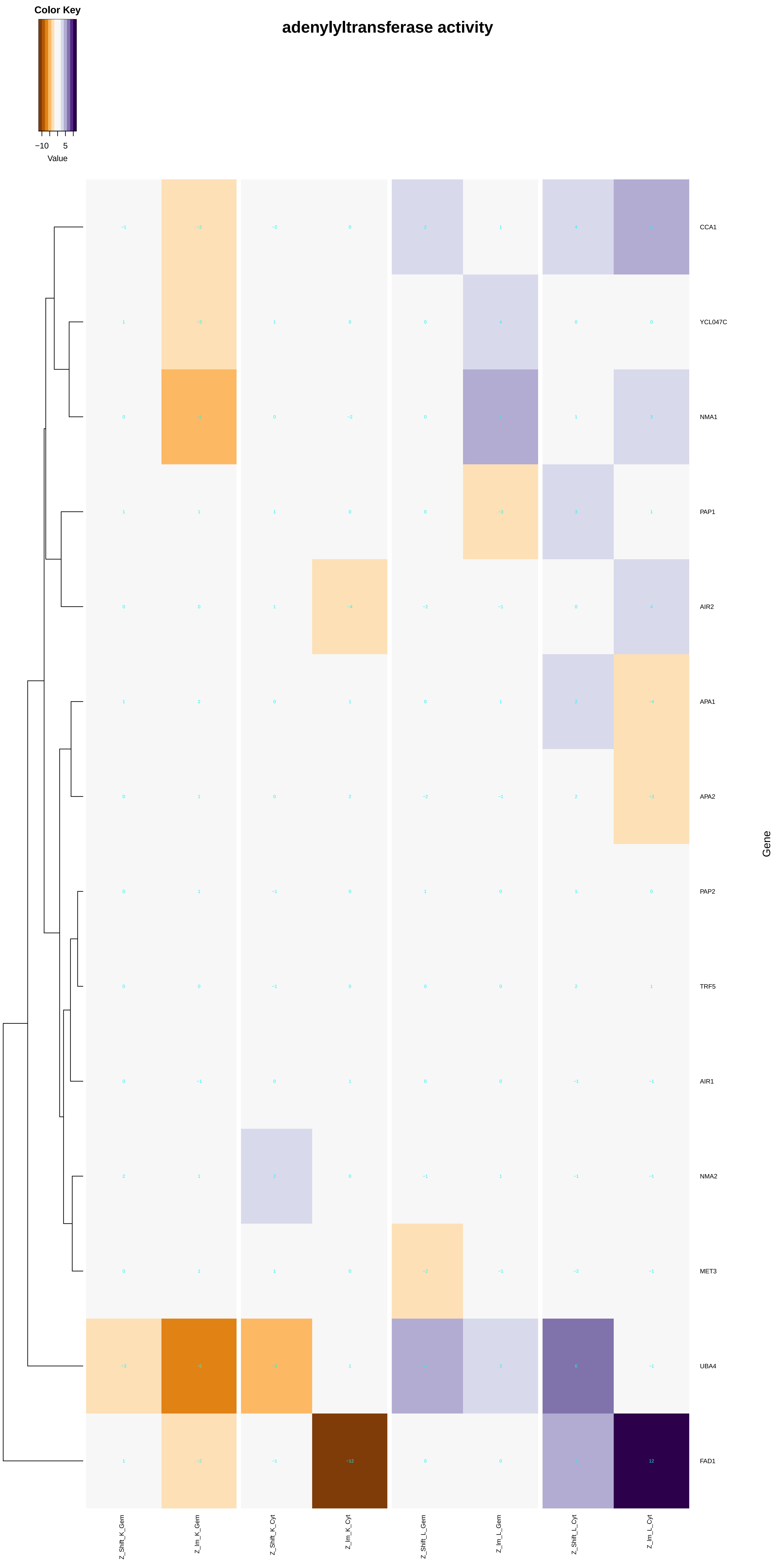
adenylyltransferase activity
Color Key
−10
5
Value
CCA1
YCL047C
NMA1
PAP1
AIR2
APA1
APA2
PAP2
TRF5
AIR1
NMA2
MET3
UBA4
FAD1
Gene
Z_Shift_K_Gem
Z_Im_K_Gem
Z_Shift_K_Cyt
Z_Im_K_Cyt
Z_Shift_L_Gem
Z_Im_L_Gem
Z_Shift_L_Cyt
Z_Im_L_Cyt

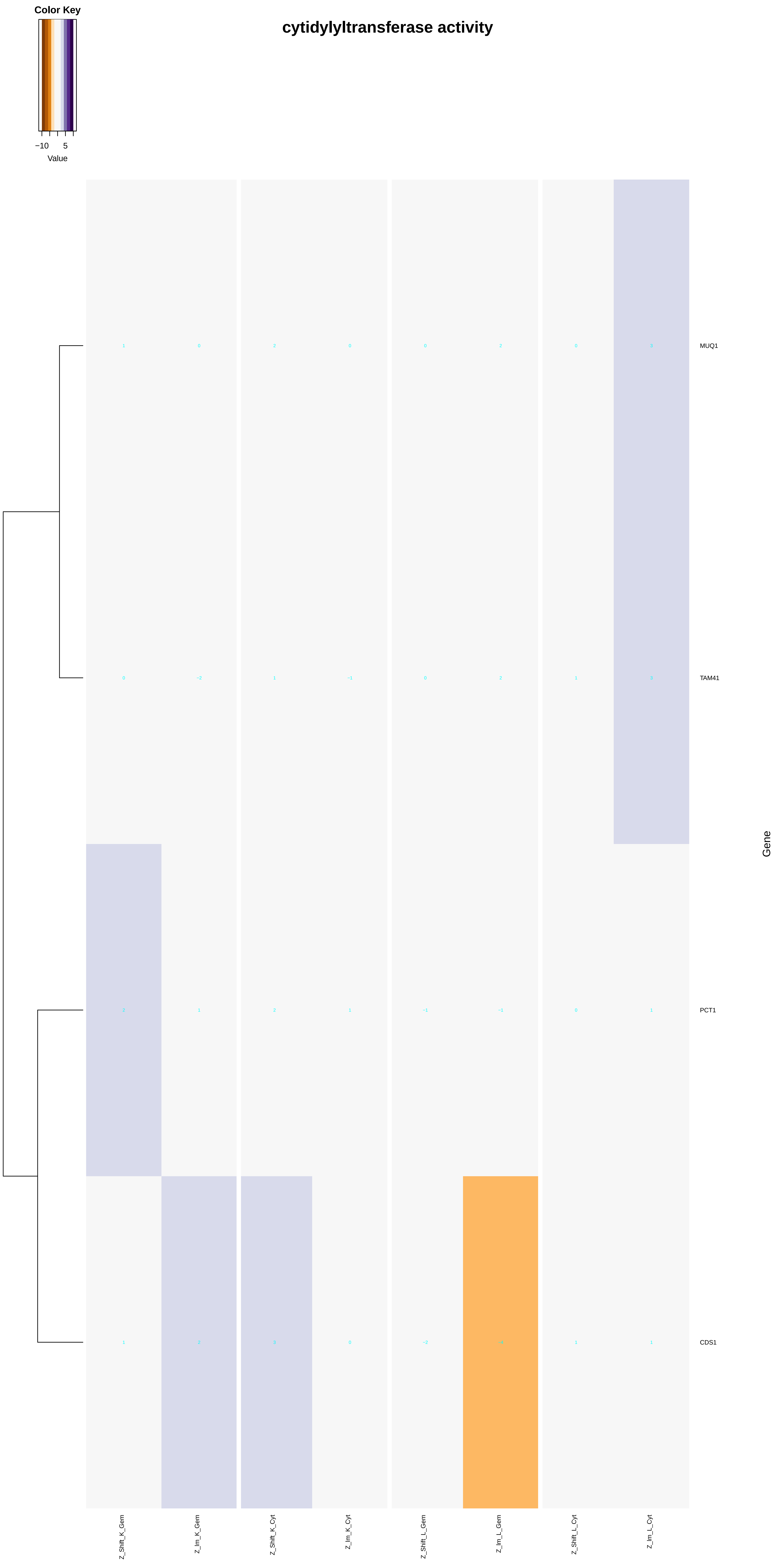
Color Key
−10
5
Value
cytidylyltransferase activity
MUQ1
TAM41
PCT1
CDS1
Gene
Z_Shift_K_Gem
Z_Im_K_Gem
Z_Shift_K_Cyt
Z_Im_K_Cyt
Z_Shift_L_Gem
Z_Im_L_Gem
Z_Shift_L_Cyt
Z_Im_L_Cyt

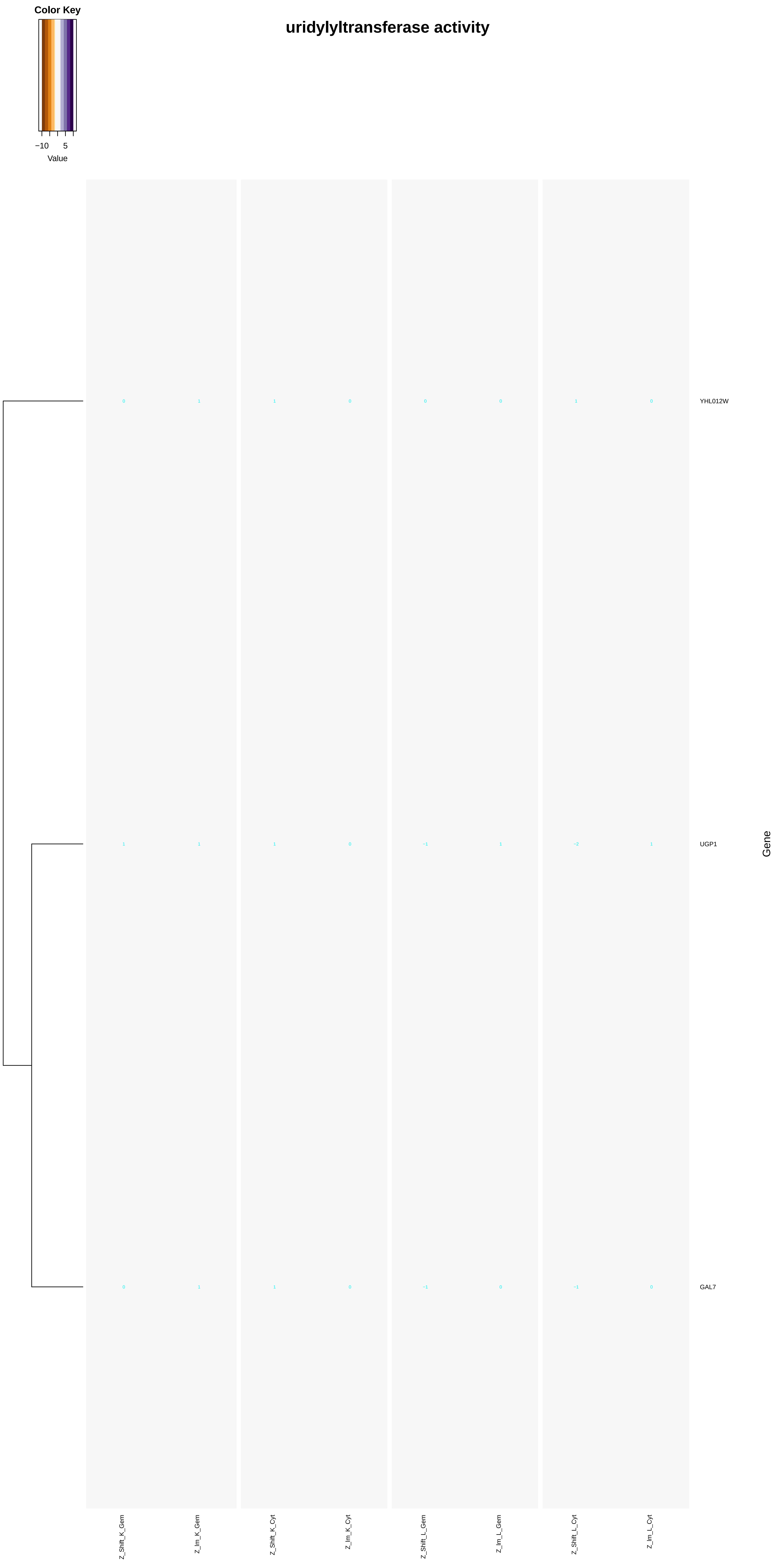

uridylyltransferase activity
Color Key
−10
5
Value
YHL012W
UGP1
GAL7
Gene
Z_Shift_K_Gem
Z_Im_K_Gem
Z_Shift_K_Cyt
Z_Im_K_Cyt
Z_Shift_L_Gem
Z_Im_L_Gem
Z_Shift_L_Cyt
Z_Im_L_Cyt

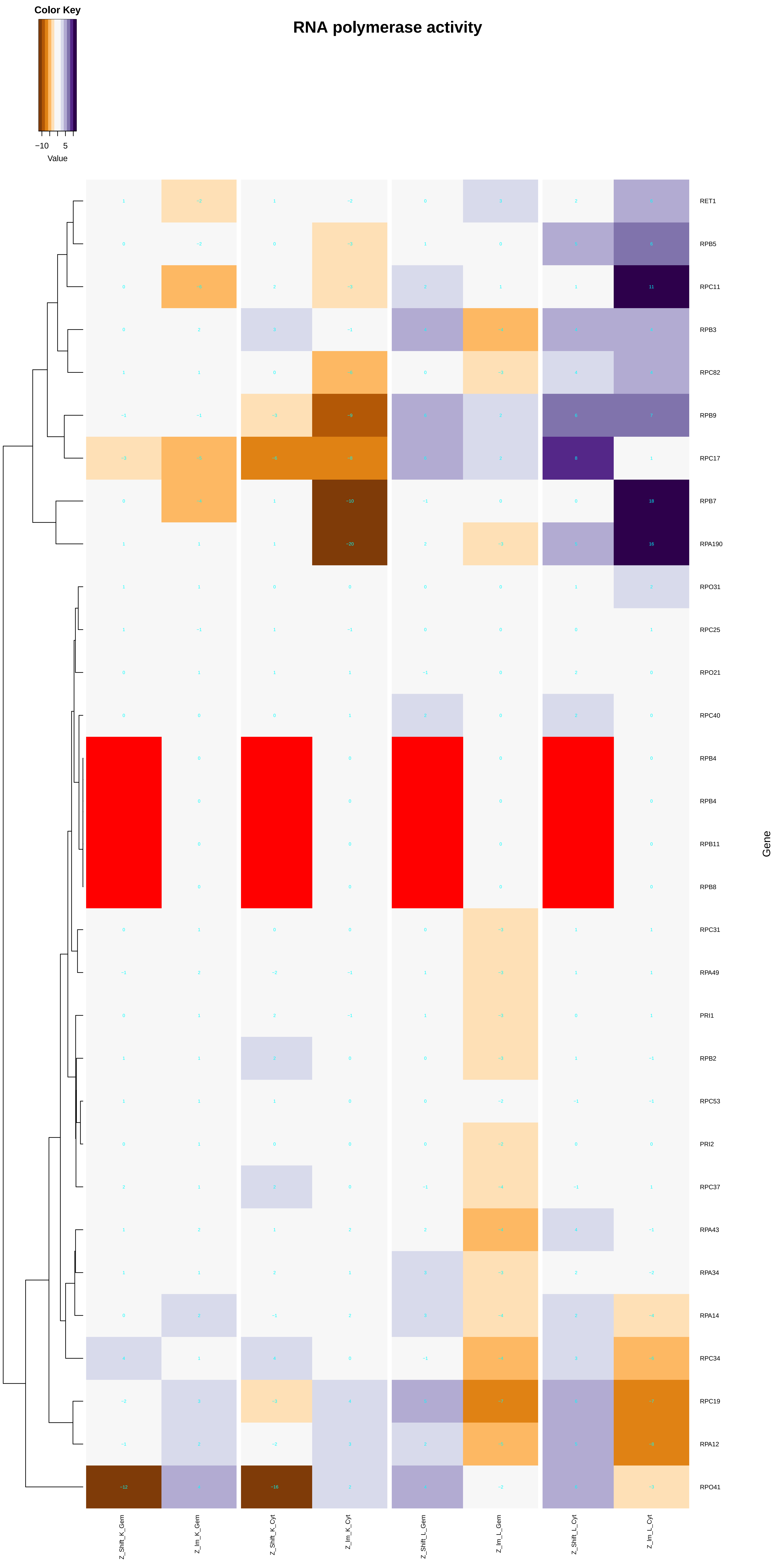
Color Key
−10
5
Value
RNA polymerase activity
RET1
RPB5
RPC11
RPB3
RPC82
RPB9
RPC17
RPB7
RPA190
RPO31
RPC25
RPO21
RPC40
RPB4
RPB4
RPB11
RPB8
RPC31
RPA49
PRI1
RPB2
RPC53
PRI2
RPC37
RPA43
RPA34
RPA14
RPC34
RPC19
RPA12
RPO41
Gene
Z_Shift_K_Gem
Z_Im_K_Gem
Z_Shift_K_Cyt
Z_Im_K_Cyt
Z_Shift_L_Gem
Z_Im_L_Gem
Z_Shift_L_Cyt
Z_Im_L_Cyt

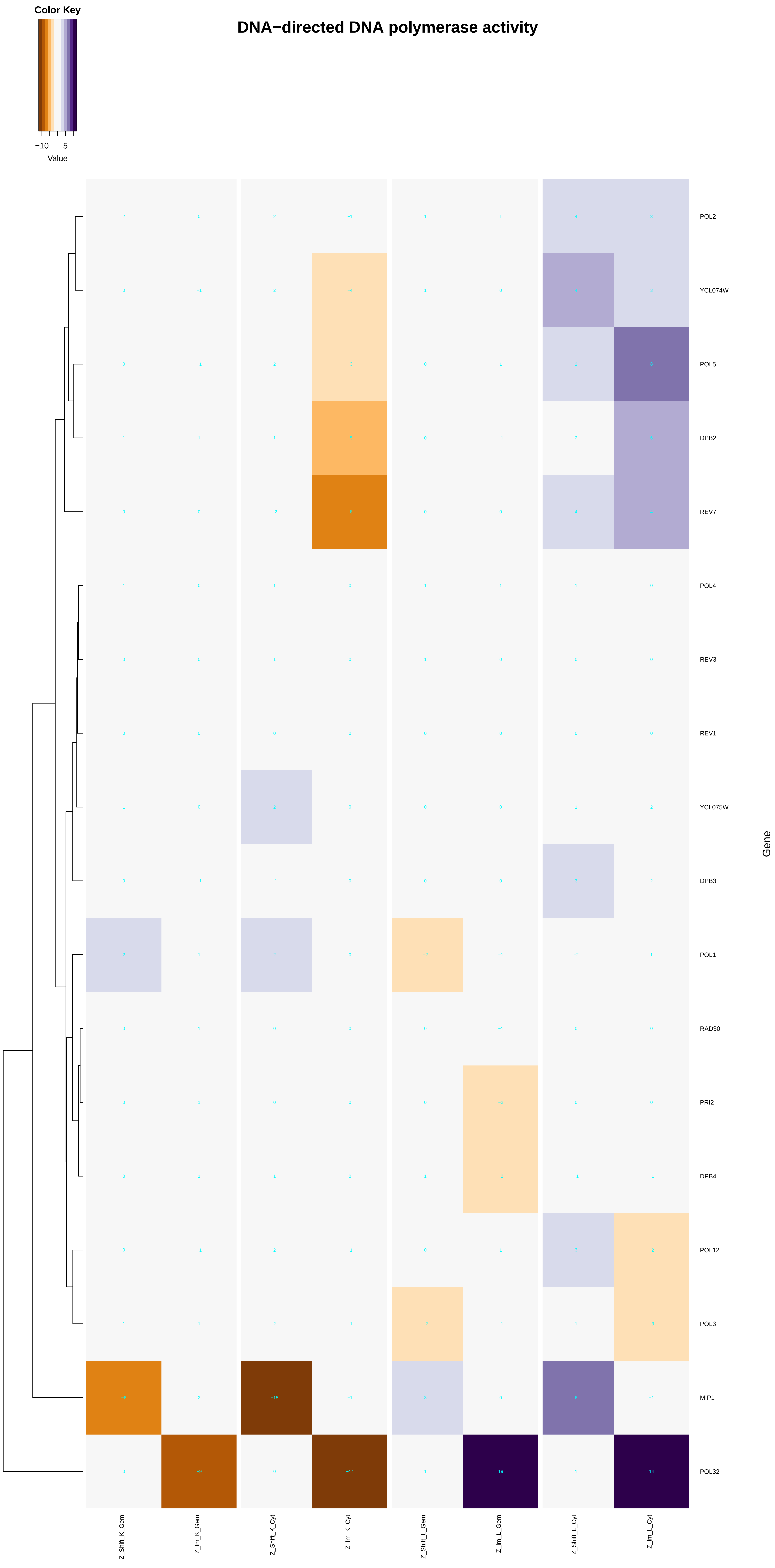
DNA−directed DNA polymerase activity
Color Key
−10
5
Value
POL2
YCL074W
POL5
DPB2
REV7
POL4
REV3
REV1
YCL075W
DPB3
POL1
RAD30
PRI2
DPB4
POL12
POL3
MIP1
POL32
Gene
Z_Shift_K_Gem
Z_lm_K_Gem
Z_Shift_K_Cyt
Z_lm_K_Cyt
Z_Shift_L_Gem
Z_lm_L_Gem
Z_Shift_L_Cyt
Z_lm_L_Cyt

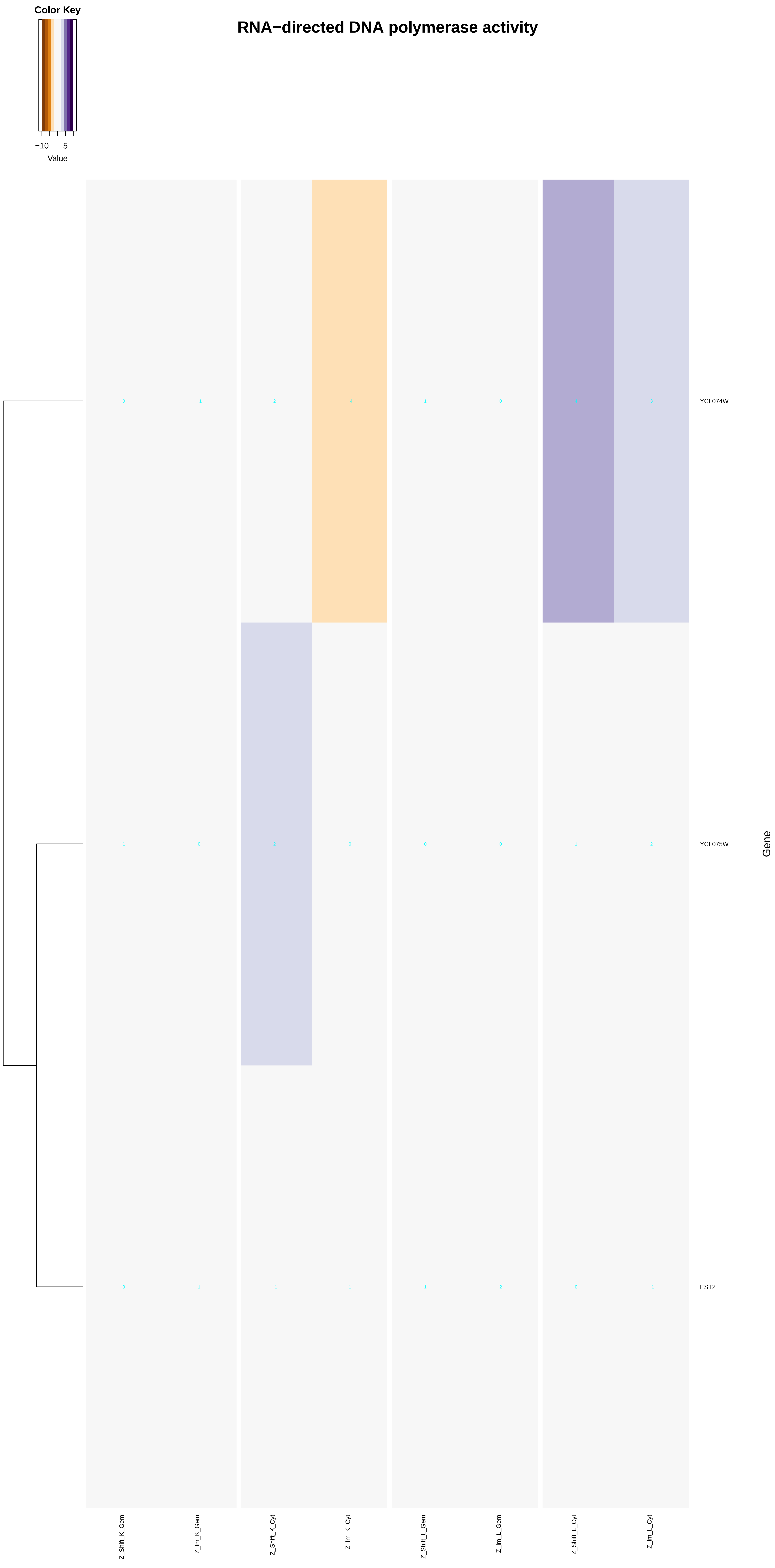
RNA−directed DNA polymerase activity
Color Key
−10
5
Value
YCL074W
YCL075W
EST2
Gene
Z_Shift_K_Gem
Z_Im_K_Gem
Z_Shift_K_Cyt
Z_Im_K_Cyt
Z_Shift_L_Gem
Z_Im_L_Gem
Z_Shift_L_Cyt
Z_Im_L_Cyt

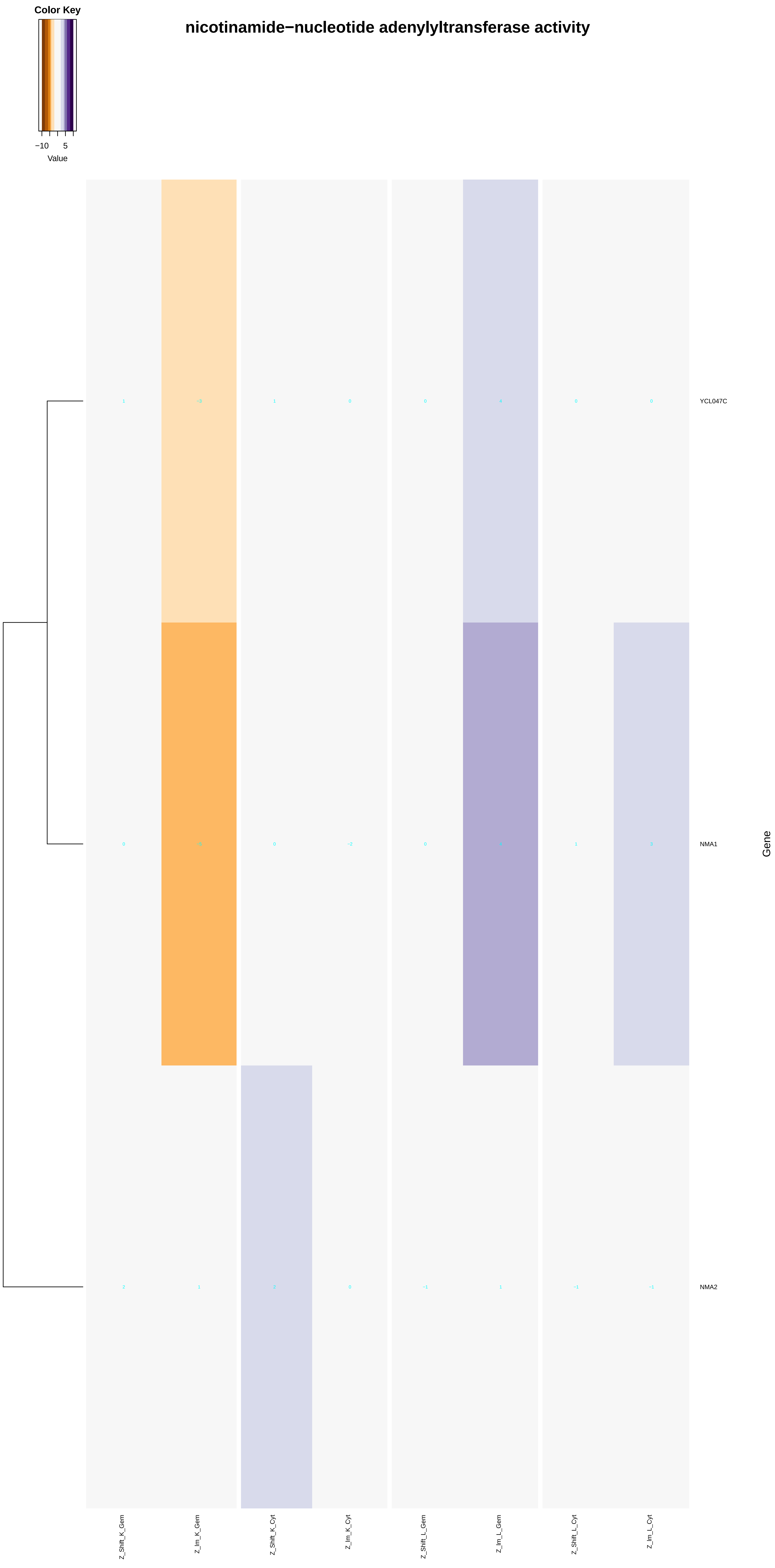

Color Key
−10
5
Value
nicotinamide−nucleotide adenylyltransferase activity
YCL047C
NMA1
NMA2
Gene
1
−3
1
0
0
4
0
0
0
−5
0
−2
0
1
3
2
1
2
0
−1
1
−1
−1
Z_Shift_K_Gem
Z_Im_K_Gem
Z_Shift_K_Cyt
Z_Im_K_Cyt
Z_Shift_L_Gem
Z_Im_L_Gem
Z_Shift_L_Cyt
Z_Im_L_Cyt

polynucleotide adenylyltransferase activity

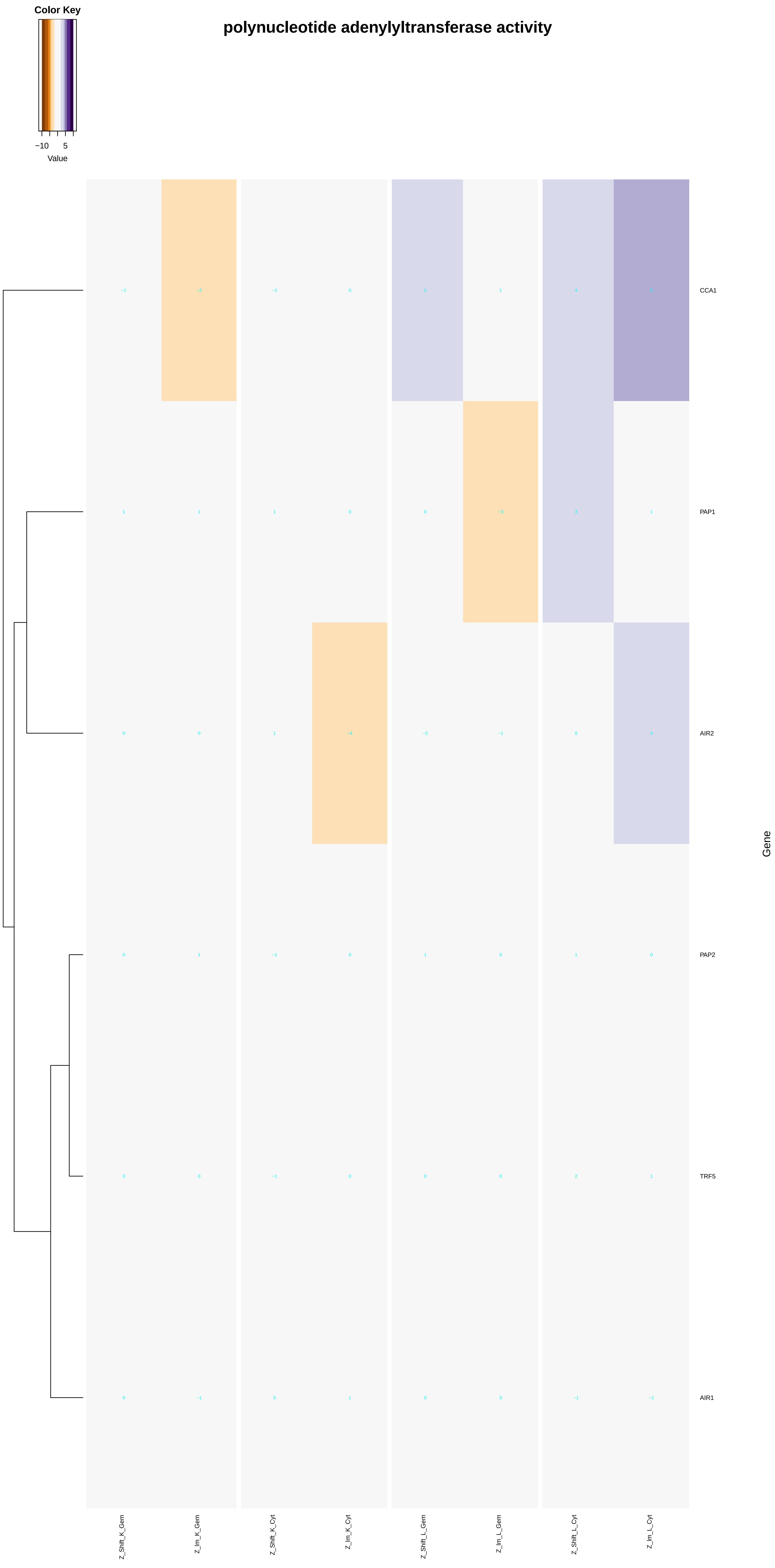

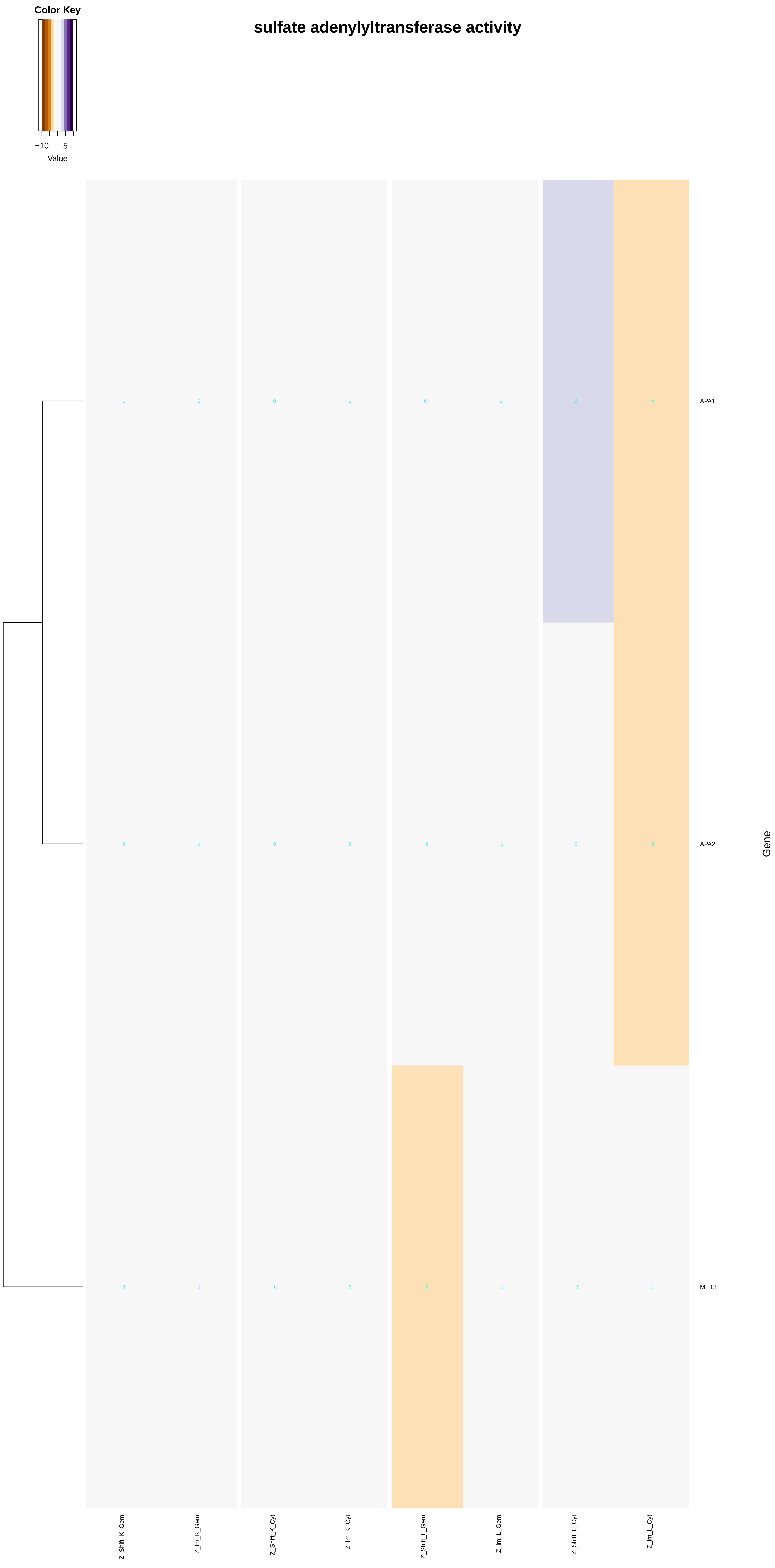
sulfate adenylyltransferase activity
Color Key
−10
5
Value
APA1
APA2
MET3
Gene
Z_Shift_K_Gem
Z_Im_K_Gem
Z_Shift_K_Cyt
Z_Im_K_Cyt
Z_Shift_L_Gem
Z_Im_L_Gem
Z_Shift_L_Cyt
Z_Im_L_Cyt

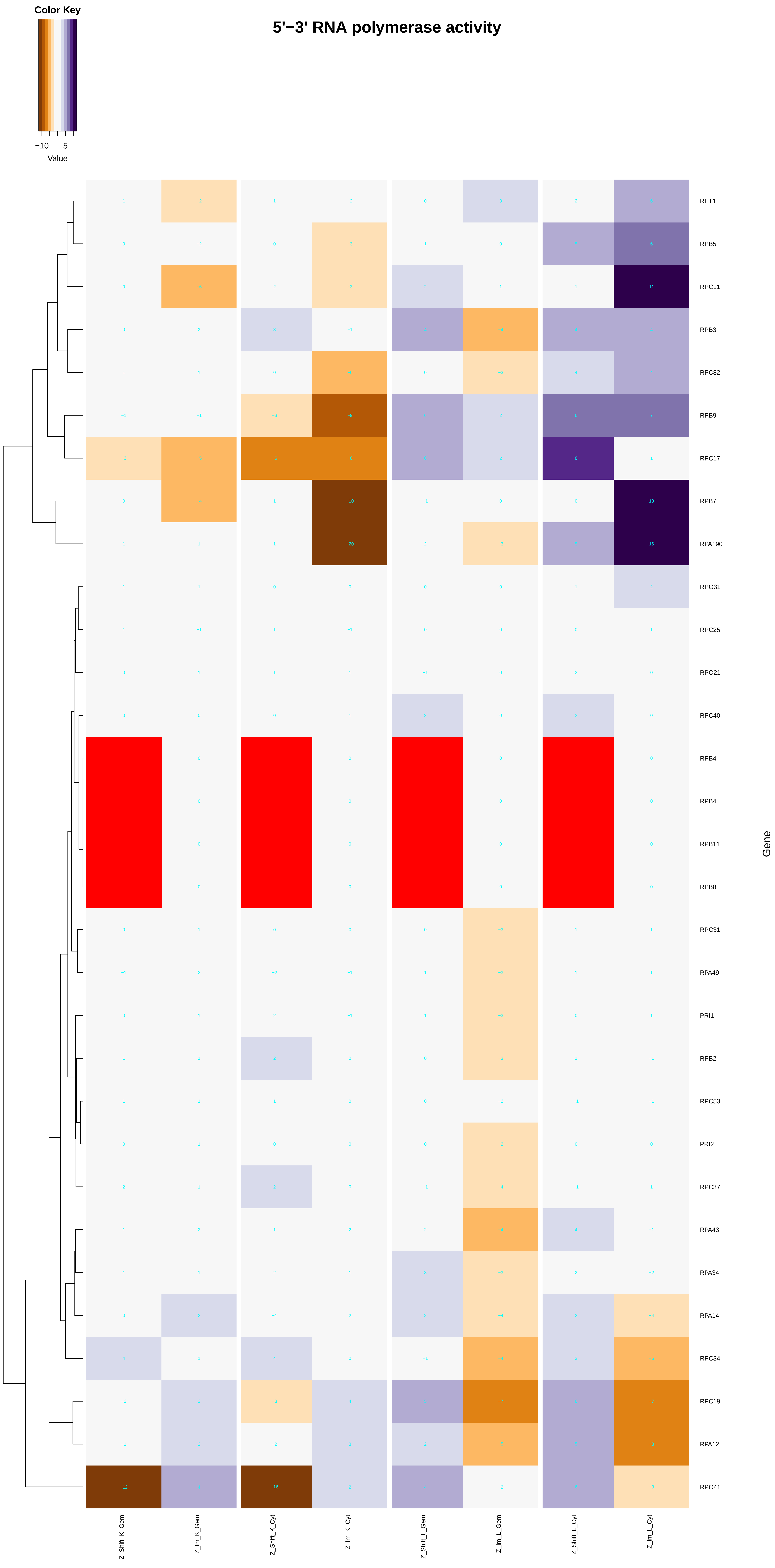

Color Key
−10
5
Value
5'−3' RNA polymerase activity
RET1
RPB5
RPC11
RPB3
RPC82
RPB9
RPC17
RPB7
RPA190
RPO31
RPC25
RPO21
RPC40
RPB4
RPB4
RPB11
RPB8
RPC31
RPA49
PRI1
RPB2
RPC53
PRI2
RPC37
RPA43
RPA34
RPA14
RPC34
RPC19
RPA12
RPO41
Gene
Z_Shift_K_Gem
Z_Im_K_Gem
Z_Shift_K_Cyt
Z_Im_K_Cyt
Z_Shift_L_Gem
Z_Im_L_Gem
Z_Shift_L_Cyt
Z_Im_L_Cyt

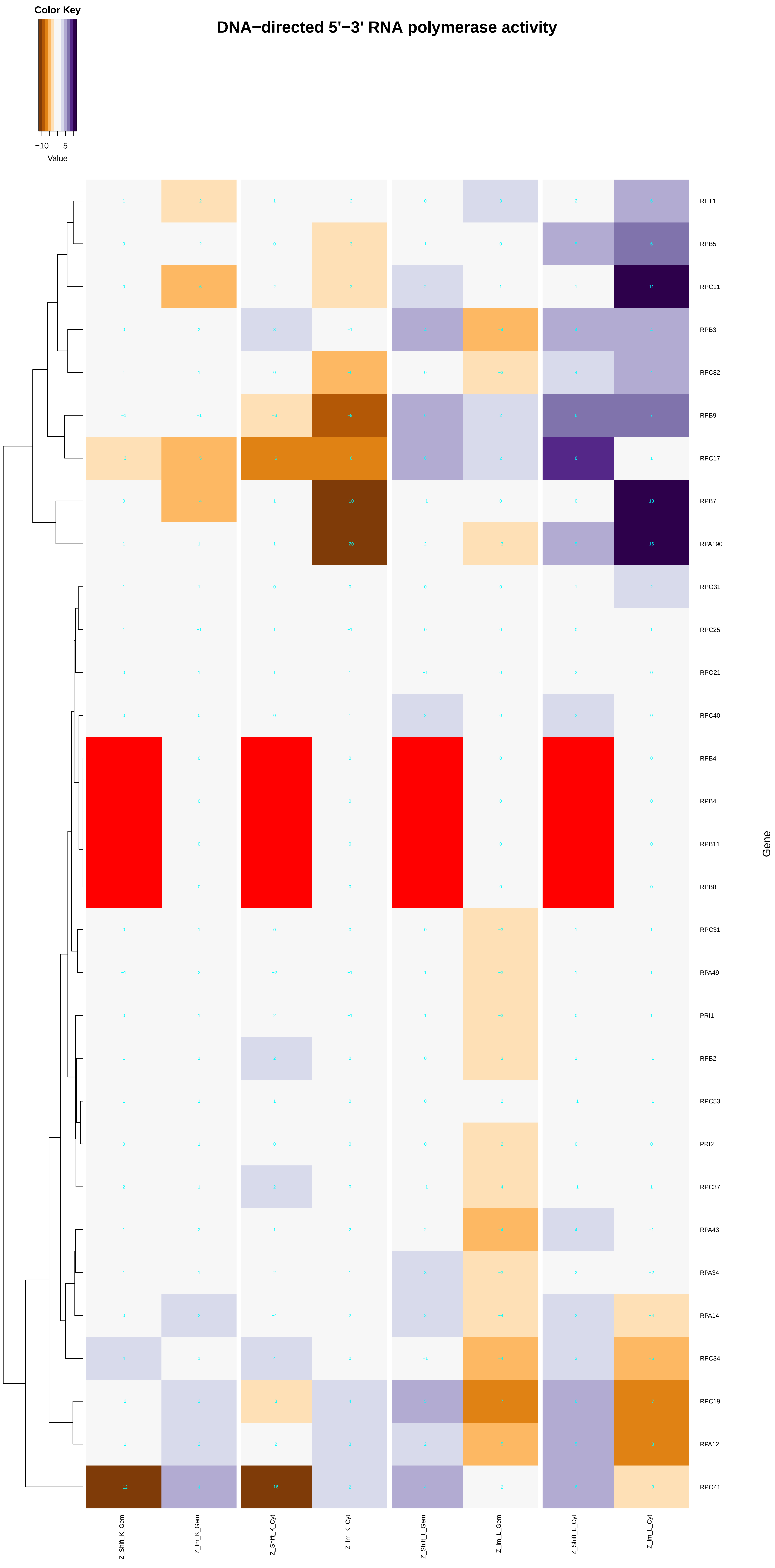

Color Key
−10
5
Value
DNA−directed 5'−3' RNA polymerase activity
RET1
RPB5
RPC11
RPB3
RPC82
RPB9
RPC17
RPB7
RPA190
RPO31
RPC25
RPO21
RPC40
RPB4
RPB4
RPB11
RPB8
RPC31
RPA49
PRI1
RPB2
RPC53
PRI2
RPC37
RPA43
RPA34
RPA14
RPC34
RPC19
RPA12
RPO41
Gene
Z_Shift_K_Gem
Z_Im_K_Gem
Z_Shift_K_Cyt
Z_Im_K_Cyt
Z_Shift_L_Gem
Z_Im_L_Gem
Z_Shift_L_Cyt
Z_Im_L_Cyt

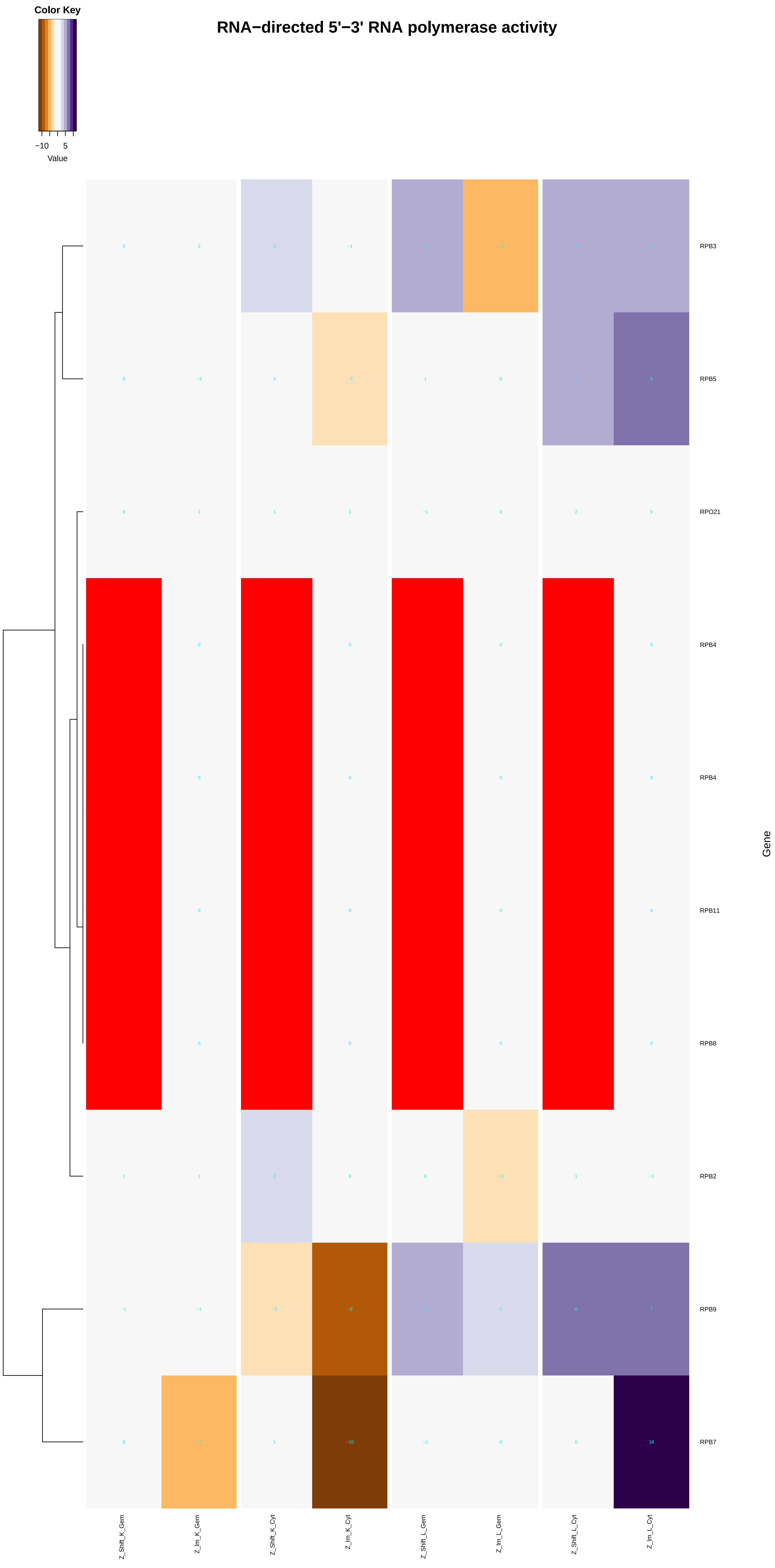
Color Key
−10
5
Value
RNA−directed 5'−3' RNA polymerase activity
RPB3
RPB5
RPO21
RPB4
RPB4
RPB11
RPB8
RPB2
RPB9
RPB7
Gene
Z_Shift_K_Gem
Z_Im_K_Gem
Z_Shift_K_Cyt
Z_Im_K_Cyt
Z_Shift_L_Gem
Z_Im_L_Gem
Z_Shift_L_Cyt
Z_Im_L_Cyt

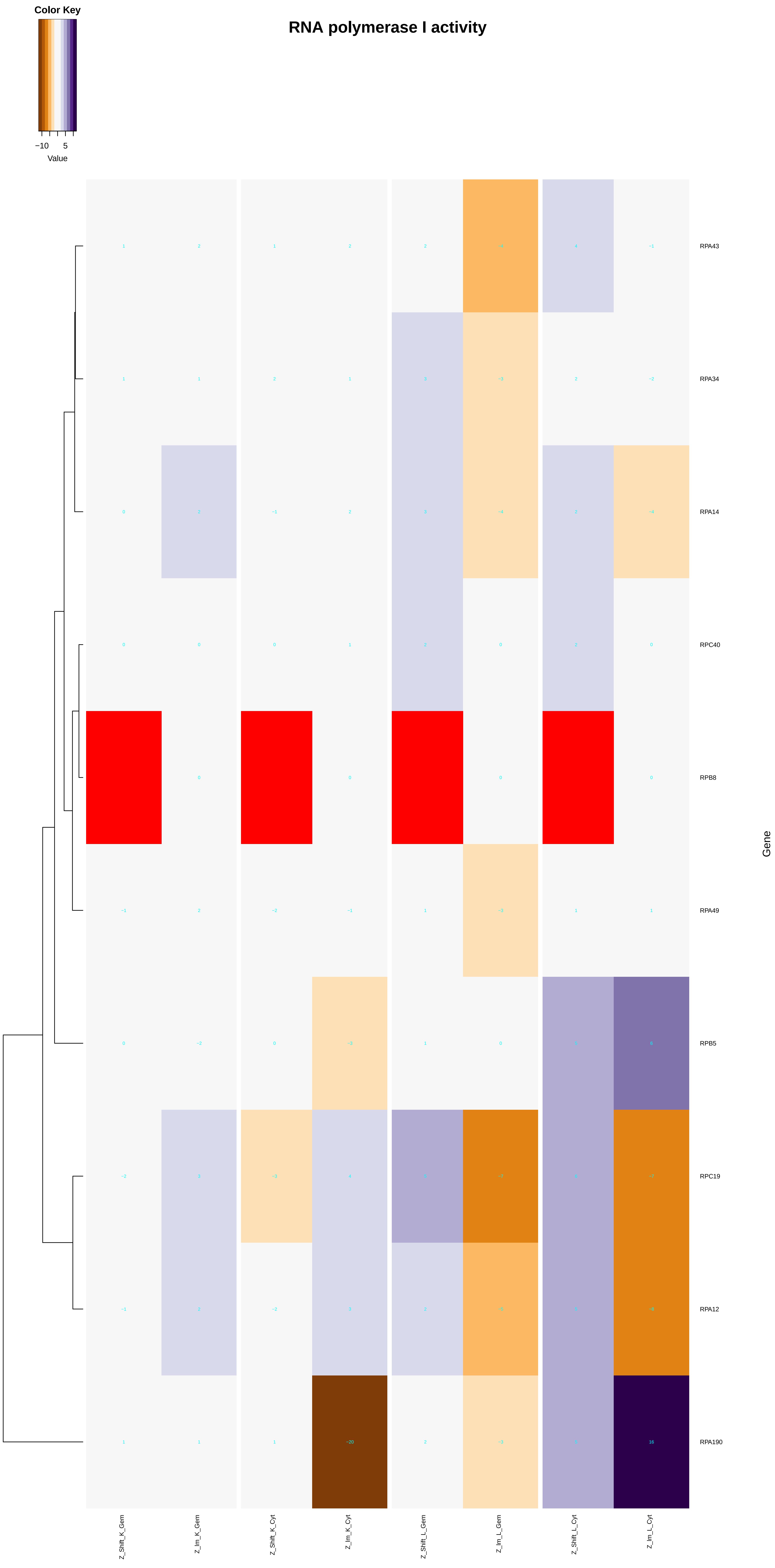

Color Key
−10
5
Value
RNA polymerase I activity
RPA43
RPA34
RPA14
RPC40
RPB8
RPA49
RPB5
RPC19
RPA12
RPA190
Gene
Z_Shift_K_Gem
Z_Im_K_Gem
Z_Shift_K_Cyt
Z_Im_K_Cyt
Z_Shift_L_Gem
Z_Im_L_Gem
Z_Shift_L_Cyt
Z_Im_L_Cyt

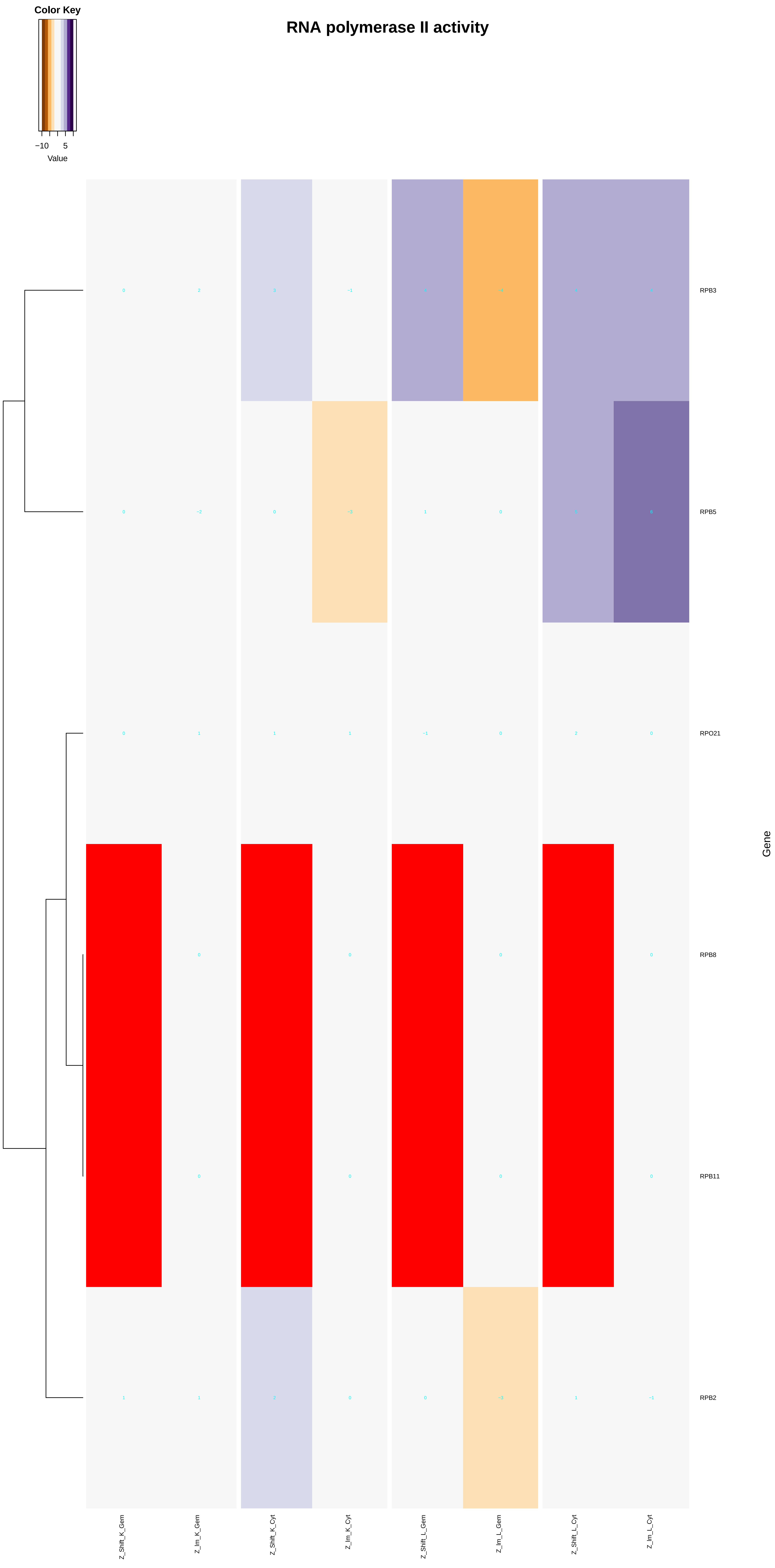

Color Key
−10 5
Value
RNA polymerase II activity
RPB3
RPB5
RPO21
RPB8
RPB11
RPB2
Gene
Z_Shift_K_Gem
Z_Im_K_Gem
Z_Shift_K_Cyt
Z_Im_K_Cyt
Z_Shift_L_Gem
Z_Im_L_Gem
Z_Shift_L_Cyt
Z_Im_L_Cyt

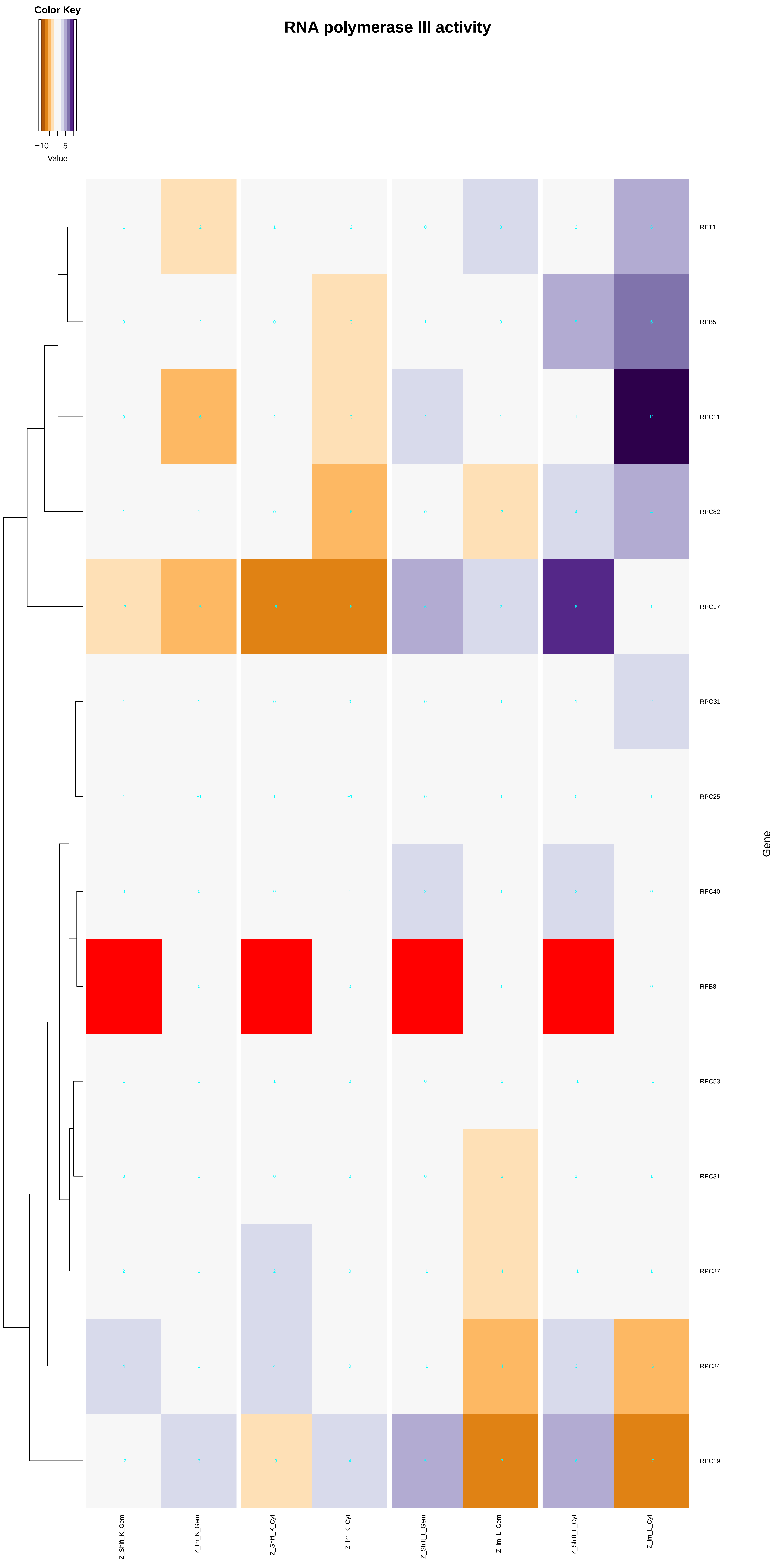
RNA polymerase III activity
Color Key
−10
5
Value
RET1
RPB5
RPC11
RPC82
RPC17
RPO31
RPC25
RPC40
RPB8
RPC53
RPC31
RPC37
RPC34
RPC19
Gene
Z_Shift_K_Gem
Z_Im_K_Gem
Z_Shift_K_Cyt
Z_Im_K_Cyt
Z_Shift_L_Gem
Z_Im_L_Gem
Z_Shift_L_Cyt
Z_Im_L_Cyt

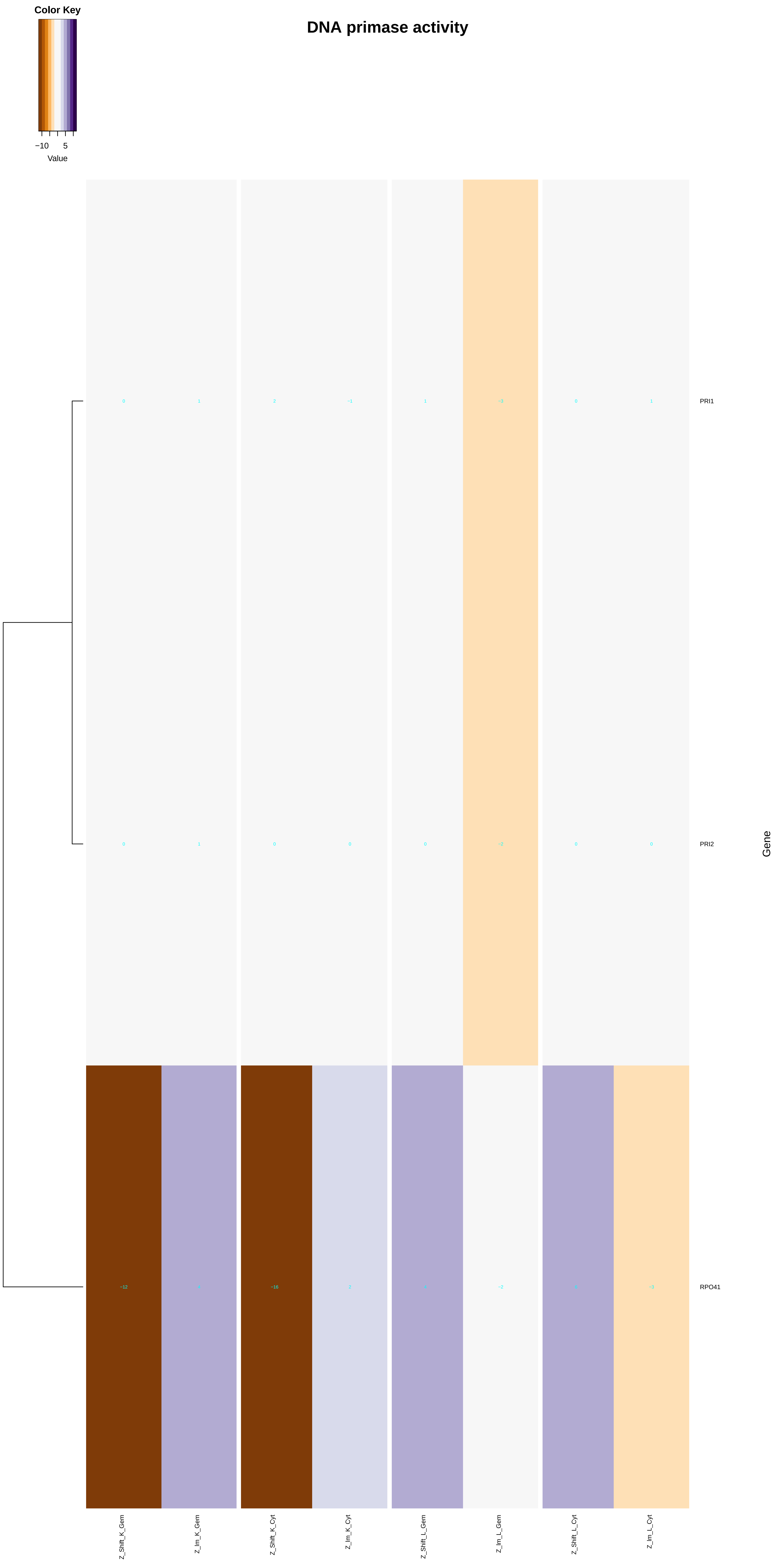

Color Key
−10
5
Value
DNA primase activity
0
1
2
−1
1
−3
0
1
PRI1
0
1
0
0
0
−2
0
0
PRI2
−12
4
−16
2
4
−2
6
−3
RPO41
Gene
Z_Shift_K_Gem
Z_Im_K_Gem
Z_Shift_K_Cyt
Z_Im_K_Cyt
Z_Shift_L_Gem
Z_Im_L_Gem
Z_Shift_L_Cyt
Z_Im_L_Cyt
